# The impact of increasing the availability of lower energy foods for home delivery and socio-economic position: a randomised control trial examining effects on meal energy intake and later energy intake

**DOI:** 10.1017/S0007114522002197

**Published:** 2023-04-14

**Authors:** Tess Langfield, Andrew Jones, Eric Robinson

**Affiliations:** Department of Psychological Sciences, University of Liverpool, Liverpool L69 7ZA, UK

**Keywords:** Socio-economic status, Socio-economic position, Availability, Food environment, Energy intake

## Abstract

Increasing the availability of lower energy food options is a promising public health approach. However, it is unclear the extent to which availability interventions may result in consumers later ‘compensating’ for reductions in energy intake caused by selecting lower energy food options and to what extent these effects may differ based on socio-economic position (SEP). Our objective was to examine the impact of increasing availability of lower energy meal options on immediate meal energy intake and subsequent energy intake in participants of higher *v*. lower SEP. In a within-subjects design, seventy-seven UK adults ordered meals from a supermarket ready meal menu with standard (30 %) and increased (70 %) availability of lower energy options. The meals were delivered to be consumed at home, with meal intake measured using the Digital Photography of Foods Method. Post-meal compensation was measured using food diaries to determine self-reported energy intake after the meal and the next day. Participants consumed significantly less energy (196 kcal (820 kJ), 95 % CI 138, 252) from the menu with increased availability of lower energy options *v*. the standard availability menu (*P* < 0·001). There was no statistically significant evidence that this reduction in energy intake was substantially compensated for (33 % compensated, *P* = 0·57). The effects of increasing availability of lower energy food items were similar in participants from lower and higher SEP. Increasing the availability of lower energy food options is likely to be an effective and equitable approach to reducing energy intake which may contribute to improving diet and population health.

The high prevalence of obesity in most developed countries is likely to have been impacted by changes to the food environment^(1,2)^ and, in particular, the widespread availability of energy-dense food and drink products served in large portion sizes^(3,4)^. Therefore, it is now widely recognised that changes to the structure of the food environment are needed to reduce population-level energy intake and obesity^(1)^. However, because diet and obesity are socio-economically patterned, whereby lower socio-economic position (SEP) is associated with an increased risk of higher BMI and obesity^(5,6)^, it is imperative that interventions designed to address the food environment do not further widen SEP inequalities in obesity.

One intervention approach that targets the structure of the food environment is to increase the relative availability of lower energy food options (i.e. by increasing the proportion of food items available that are lower in kcals). Increasing availability of lower energy options may have an equitable effect on diet because unlike other types of intervention (e.g. information provision interventions), it is less reliant on consumers being motivated or able to consciously change their behaviour^(7,8)^. However, the extent to which increasing availability of lower energy options has an equitable effect on the diet of lower and higher SEP individuals has received some but limited empirical testing^(9)^. While studies to date have found that the effects of increasing availability of healthier foods on food selection are not statistically moderated by participant SEP^(10–14)^, testing has been limited to a small number of studies that have predominantly used hypothetical food choice which does not require participants to select and consume actual meals.

A further limitation of studies examining availability interventions is that none we are aware of have examined the impact of increasing availability of lower energy options at a meal on energy intake beyond that meal. Because controlled nutrition experiments indicate that consuming less energy at a meal will sometimes result in later ‘compensation’ (i.e. consuming more energy later in the day)^(15,16)^ and the selection of a lower energy meal may make some consumers feel that they are licensed to ‘overeat’ later in the day^(17)^, the overall effect that increasing the availability of lower energy options has on diet is currently unclear. Furthermore, recent research indicates that SEP may affect the likelihood of compensatory eating. In particular, it has been hypothesised that because lower SEP is associated with experiencing food scarcity and insecurity, this may result in a greater drive to avoid periods of lower energy intake^(18)^. In line with this, studies have found that lower perceived SEP (observed or experimentally manipulated) is associated with an increased sensitivity to the energy content of foods^(19)^ and an increased likelihood of eating beyond energy needs after consuming a lower energy meal^(20)^. Therefore, there is a need to understand the effect that increasing the availability of lower energy meal options has on acute (i.e. at that meal) and subsequent (i.e. after that meal) energy intake in both lower and higher SEP groups.

In the present study, we examined the effect of increasing the availability of lower energy meal options on meal energy intake and subsequent 24-h energy intake in participants of lower *v*. higher SEP. We primarily based SEP on highest achieved education level to be consistent with existing literature^(13,14,21)^ and because education level is reliably related to diet quality and obesity^(22,23)^. In line with an increasing trend in use of online food delivery services in the UK^(24)^, participants made supermarket ready meal choices using an online food ordering platform and meals were home delivered for consumption.

## Methods

### Participants

Participants were recruited between April and July 2021. Eligibility criteria were as follows: UK residents aged 18 years or over, fluent in English, access to Internet, a camera (e.g. camera phone) and a microwave/oven to prepare ready meals at home, no current or history of eating disorders, not on medication affecting appetite, no dietary restrictions (e.g. vegetarian), no history of food allergy or anaphylaxis. Potential participants were recruited using social media posts (Facebook, Instagram, Twitter), participant mailing lists and from staff/students at the University of Liverpool. The study was described as investigating food choices, personality and mood (cover story). To be broadly representative of the UK adult, population recruitment was stratified by sex (50 % men, women) and student status (3·5 % yes). As education level was our primary measure of SEP, we recruited 50 % of the sample to be lower (up to A level or equivalent) and 50 % higher (above A level – which equates typically to University/College level and above in the USA) SEP.

This study was conducted according to the guidelines laid down in the Declaration of Helsinki, and all procedures involving human participants were approved by the Central University Ethics Committee at the University of Liverpool (reference 8710). Written informed consent was obtained from all participants.

### Design

The study used a 2 (within-subjects: control menu *v*. increased availability menu) × 2 (between-subjects: lower SEP *v*. higher SEP) mixed design. Participants were randomised to one of two menu order conditions with randomisation stratified by sex and SEP, using the Microsoft Excel (rand()) function. Participants selected a main (from ten options) and side (from six options) from two menus which consisted of popular items from a UK supermarket (Tesco). Lower and higher energy mains and sides were defined as ≤400 kcal (1674 kJ) *v*. >400 kcal (1674 kJ) and ≤300 kcal (1255 kJ) *v*. >300 kcal (1255 kJ), respectively, based on the energy content of the range of products available and Public Health England guidance on main meal energy intake^(25)^. The distribution of lower *v*. higher energy options in the control menu condition was representative of the supermarket’s online stock when the study was conducted; 3/10 mains and 2/6 sides were lower energy options. Proportions were reversed in the increased availability menu (7/10 mains and 4/6 sides were lower energy), with the lowest and highest energy meal items retained across both menus. See online Supplementary Materials for details about menu design, menu items and nutritional information.

### Participant measures

#### Socio-economic position

Highest educational qualification was the primary measure of SEP. Consistent with other research^(26)^, participants with A levels or less were categorised as lower in SEP, and those with above A levels (e.g. degree level) were higher in SEP. Participants also reported their number of years in higher education, household income and subjective social status as additional measures of SEP for use in sensitivity analyses. See online Supplementary Materials for questionnaire items in full.

#### Demographic and individual difference measures

Participants’ self-reported demographic (sex, age, ethnicity, employment status) and personal (physical activity days in last week, frequency of ready meal consumption, weight, height) characteristics were collected. Participants also completed eating behaviour individual difference measures that we reasoned could potentially moderate the effect of increased availability on meal energy intake. The food choice questionnaire^(27)^ subscales relating to ‘Health’ (six items, e.g. ‘Keeps me healthy’) and ‘Weight control’ (three items, e.g. ‘Is low in calories’) motives were included. Satiety responsiveness was measured using the four-item satiety responsiveness subscale of the Adult Eating Behaviour Questionnaire^(28)^. To assess tendencies to plate clear, we used the five-item plate-clearing tendencies scale^(29,30)^, and food waste concerns were assessed using the five-item food waste concerns scale^(31)^.

### Study outcome measures

The primary outcome measures were total meal energy selected (i.e. kcal content of selected main plus side) and total meal energy consumed (in kcals). The latter was calculated by coding photographs taken of plate and packaging before and after consumption of each meal, using the Digital Photography of Foods Method^(32)^. Two independent coders assessed images to estimate the percentage of each food item consumed using reference images at 10 % intervals (0–100 %). Percentage consumed was then transformed based on the energy content of food items. Between the two coders, 80·4 % of coding was identical or within 10 %. For coding that was inconsistent, a third coder checked the images to resolve the difference (i.e. agreement with either coder or average between two when unclear). The secondary outcome was total later energy intake. Participants completed a dietary recall for food and drink consumption after the study meal and up to midnight the following day: Myfood24 – a validated online assessment tool for measuring self-reported 24-h energy intake^(33)^.

### Procedure

Prior to consenting to the study, participants were made aware that the study would involve selecting food items that would be delivered to their home to be eaten as evening meals. Participants did not pay for the meals or meal delivery. After providing consent, participants accessed an online survey portal and answered demographic questions and filler mood questions. Participants then selected a main and side from each menu consecutively (order of control *v*. increased availability menus randomised), with the meal options presented as images and short descriptions, and the opportunity to view additional nutritional information on request (‘Yes, I’d like to see more nutritional information’). See online Supplementary Fig. S2 for how menu options were presented to participants. Participants then rated expected liking of all menu items on a separate page. This information was collected so that a substitute item that had a similar energy content and liking could be ordered if any menu items were unavailable to be delivered. Next participants provided details to enable home delivery and completed the individual difference measures. After completing these online tasks, participants were contacted by the research team to arrange a delivery date. The meals (main and side) chosen from the control and increased availability menus were delivered together, and participants were instructed to eat the two meals on separate days (meal order determined by randomisation – same as order of menu presentation) with a 48-h washout period between meals. On the morning of delivery (herein referred to as ‘Study Day 1’), participants received a text and email reminder. On Study Day 1, participants were asked to heat meals for dinner – at the usual time they ate their evening meal – as per the instructions indicated on the packaging, to take photos of their meals (i.e. plate and packaging) before and after finishing eating, and to not share food with others. On Study Day 2, participants were sent instructions to complete the dietary recall. On Study Days 3 and 4, the same process was repeated for the participants’ second meal. Once participants had completed their study days, they were emailed a debriefing questionnaire which probed what they thought the aim of the study was (later coded by two independent researchers to identify any participants guessing the study aims), before being debriefed and compensated for their time. During the study, participant questionnaires included attention checks (e.g. ‘In the past week, how many times have you been to the moon?’) as well as consistency checks (e.g. probing highest educational qualification multiple times) to identify inconsistent/inaccurate responses.

### Sample size and statistical analysis

To detect small- to moderate-sized effects of availability menu type and moderation of the effect of availability on outcome measures by SEP, after accounting for potential attrition (about 25 %), we required eighty-eight participants (forty-four lower and forty-four higher SEP). See online Supplementary Materials for detailed power calculation information. The primary analysis was a mixed ANOVA, testing the effects of menu type (within-subjects, categorical: control *v*. increased availability), SEP (between-subjects, categorical: lower *v*. higher educational qualification) and the interaction between menu type*SEP on total meal kcal selected and consumed. In sensitivity analyses, we reran primary analyses to determine whether results remained the same after the following adjustments: removing participants who guessed the study aims, substituting the primary SEP measure with alternative measures (years in higher education, subjective social status, equivalised household income), retaining all participants who were excluded from primary analyses, controlling for menu order effects (unplanned) and excluding individuals who did not receive their preferred menu items (unplanned). The primary analysis approach was repeated with total later energy intake (secondary outcome). To account for bias in self-reported daily energy intake reporting, this secondary analysis was also repeated (unplanned) after using a conservative cut-off (defined as total daily energy intake reported as being outside of the following ranges: 500–3500 kcal (2092–14644 kJ) for females and 800–4200 kcal (3347–17573 kJ) for males^(34)^) and a more stringent self-devised cut-off (self-reported daily energy intake <50 % of daily recommended intake; 1000 kcals (4184 kJ) for females and 1250 kcal (5230 kJ) for males) to remove participants with improbable total later energy intake. If we found evidence that the effect of availability on primary outcomes was moderated by SEP, we planned to explore whether any of the individual difference measures differed between SEP group and if any moderated the effect of availability on meal energy selection and intake (secondary analyses). We computed Bayes factors for the main effects and interactions in primary analyses for total meal energy selected, consumed and total energy intake. We used default priors (*r* scale fixed effects = 0·5, *r* scale random effects = 1, *r* scale covariates = 0·353). We report BF^10^s which indicate relative support for H^1^/H^0^, with conventional cut-offs of 1–3 as anecdotal evidence for H^1^, 3–10 moderate evidence for H^1^, 10–30 strong evidence for H^1^, 30–100 very strong evidence for H^1^ and > 100 extreme evidence for H^1(35)^ with inverse values indicative of the same degree of evidence for H^0^. Frequentist analyses were conducted using SPSS version 26, and Bayes factors computed in JASP version 0.16 were used for Bayesian analyses. Level of significance for statistical tests was set at *P* < 0·05 for primary and *P* < 0·01 for secondary analyses.

## Results

**Fig. 1.**
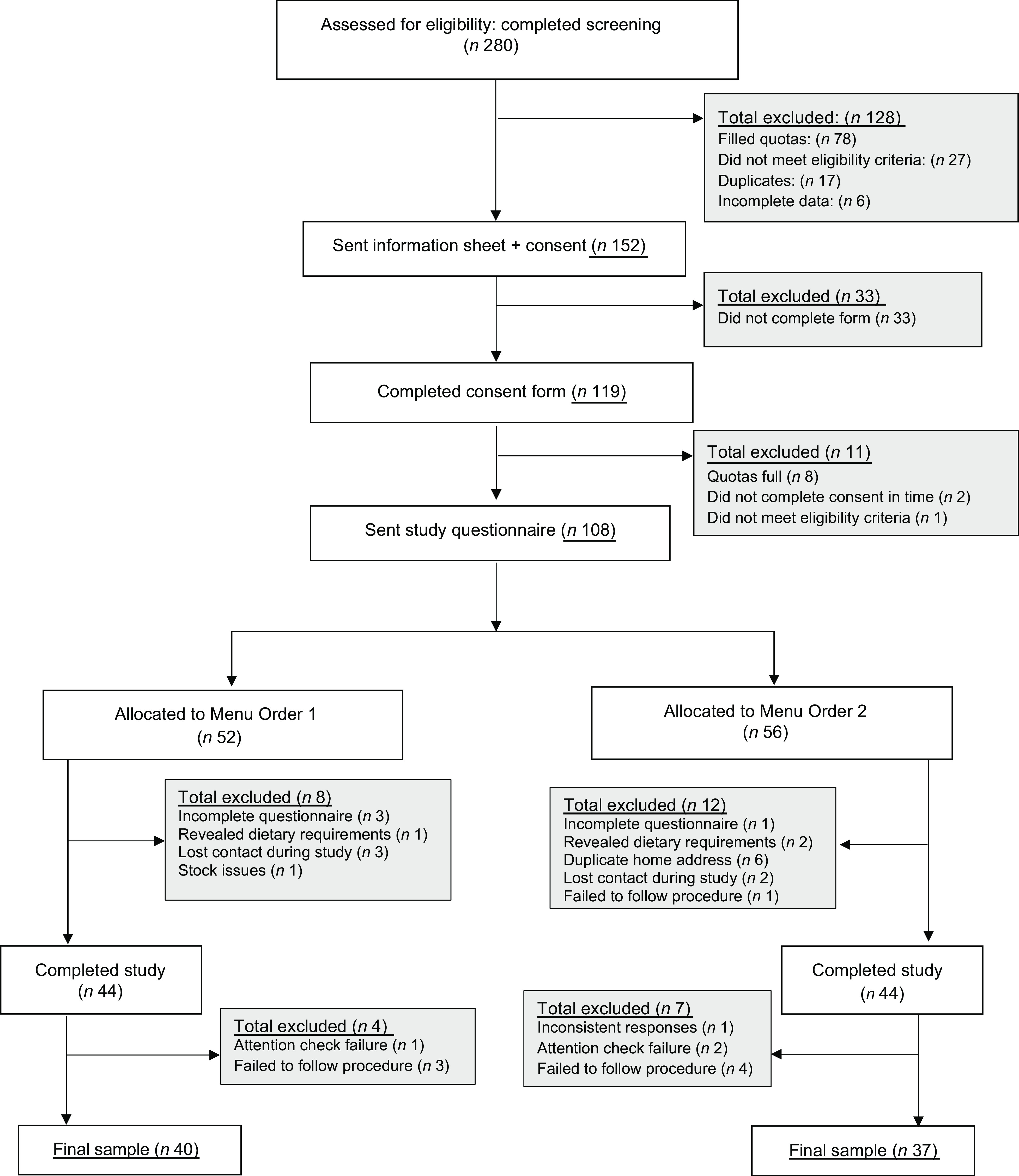
CONSORT flow chart for participant enrolment and study completion.

A total of eighty-eight participants (50 % female) completed the study. Eleven participants (12·5 %) were excluded as follows; on the basis of not following study instructions on when to eat their meals or when to complete dietary recall (*n* 4), not sending meal photos (*n* 3), inconsistent responding on highest education level at screening and in the study leading to inconsistent categorisation of higher *v*. lower SEP (*n* 1) or failing questionnaire attention checks (*n* 3), leaving a total of seventy-seven for the main analysis (see Fig. 1 for CONSORT diagram). For participant characteristics, see Table 1, and for ratings of menu items see online Supplementary Materials (higher *v*. lower SEP participants did not differ in liking of the two menu types).

**Table 1.** Summary participant characteristics by SEP group (socioeconomic position) (Numbers and percentages; mean values and standard deviations)

	Lower SEP (*n* 37)		Higher SEP (*n* 40)		Overall (*n* 77)	
	*n*	%	*n*	%	*n*	%
Sex						
Male	18	23·38	20	25·97	38	43·95
Female	19	24·68	20	25·97	39	50·65
Age						
Mean	45·35		38·30		41·69	
sd	10·69		11·32		11·51	
Ethnic group						
White	36	46·8	39	50·6	75	97·4
Mixed or multiple	1	1·3	1	1·3	2	2·6
Asian or Asian British	–	–	–	–	–	–
Black, African, Caribbean or Black British	–	–	–	–	–	–
Other ethnic group	–	–	–	–	–	–
Student or employment status						
Current student	–	–	4	5·2	4	5·2
Full or part time	26	33·8	30	39	56	72·8
Looking after home/family	4	5·2	4	5·2	8	10·4
Retired	4	5·2	2	2·6	6	7·8
Temporary or permanently sick or disabled	1	1·3	–	–	1	1·3
Unemployed/other	2	2·6	–	–	2	2·6
Highest educational qualification						
No formal qualifications	2	2·6	–	–	2	2·6
1–3 GCSE or equivalent	5	6·5	–	–	5	6·5
4 + GCSE or equivalent	16	20·8	–	–	16	20·8
A level or equivalent	14	18·2	–	–	14	18·2
Certificate of higher education (CertHE) or equivalent	–	–	5	6·5	5	6·5
Diploma of higher education (DipHE) or equivalent	–	–	4	5·2	4	5·2
Bachelor or equivalent	–	–	20	24·7	20	24·7
Master’s degree or equivalent	–	–	8	10·4	8	10·4
Doctorate or equivalent	–	–	3	3·9	3	3·9
Years in higher education						
Mean	1·22		5·45		3·42	
sd	1·37		2·14		2·79	
Equivalised household income (£)						
Mean	21 783·09		27 997·80		25 011·51	
sd	16 209·12		16 167·71		16 381·65	
Subjective socio-economic status (1–10)						
Mean	5·00		5·78		5·40	
sd	1·45		1·25		1·40	
BMI (kg/m^2^)						
Mean	33·12		28·49		30·71	
sd	9·04		5·96		7·90	
Dieting status						
Yes	6	7·8	5	6·5	11	14·3
No	31	40·3	35	45·5	66	85·8
Physical activity level (no. of days in the last week)	2·76	1·89	3·68	2·28	3·23	2·14
Ready meal consumption frequency						
Never or not in the last year	1	1·3	2	2·6	3	3·9
Less than once per month	13	16·9	13	16·9	26	33·8
1–3 times per month	19	24·7	17	22·1	36	46·8
1–2 times per week	3	3·9	7	9·1	10	13·0
3 times per week or more	1	1·3	1	1·3	2	2·6

SEP, socio-economic position.

GCSE, General Certificate of Secondary Education.

Values are mean values and standard deviations unless otherwise stated.

### Primary analyses

**Fig. 2.**
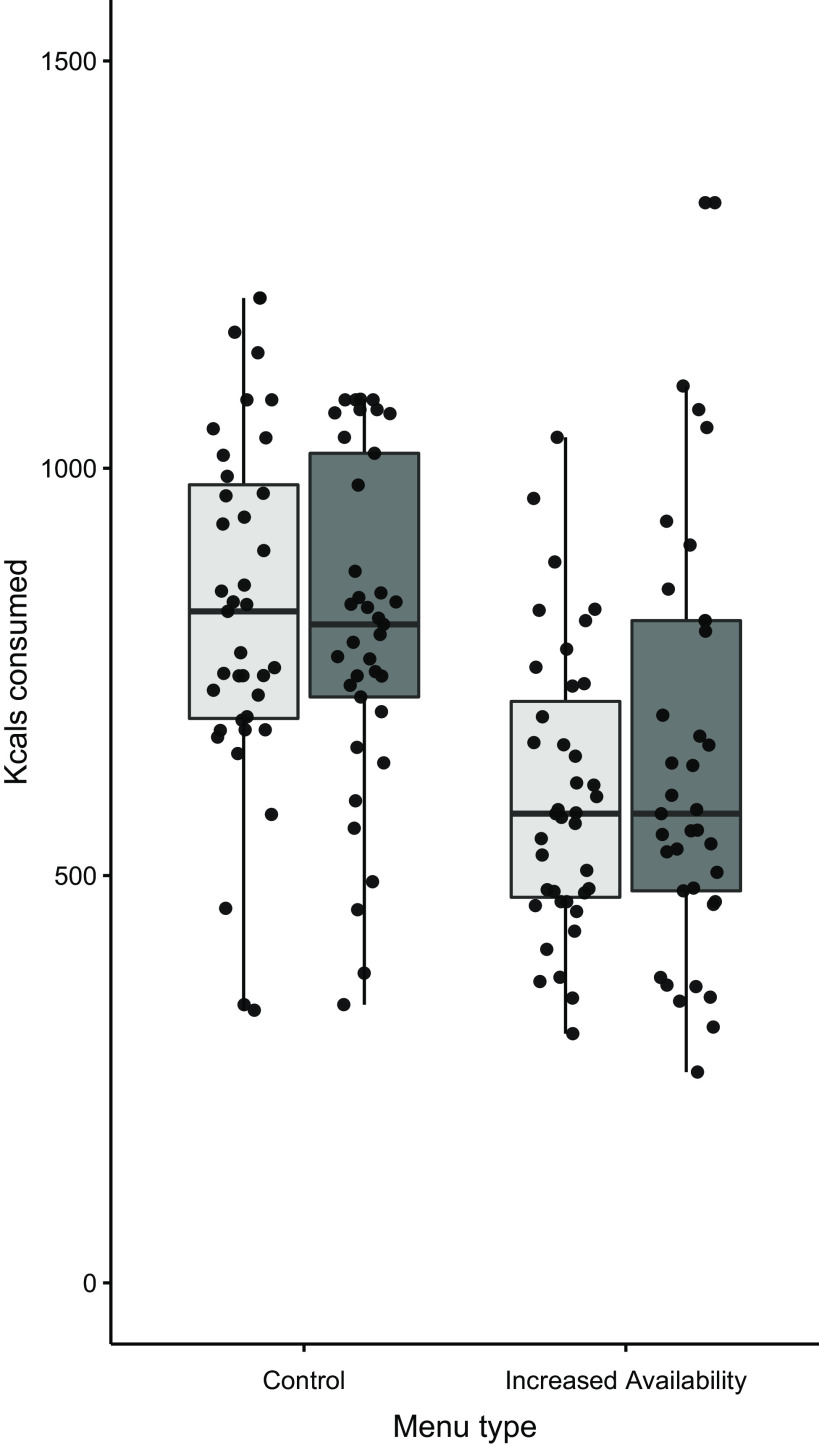
Meal energy intake (kcal) by menu condition and SEP. 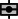, higher; 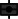, lower. SEP, socio-economic position.

As analyses examining total meal energy selected and consumed produced the same pattern of results, analyses for energy of meal selected are reported in full in the online Supplementary Materials. There were missing data (*n* 1) on energy consumed due to unclear photos taken. ANOVA revealed a main effect of menu type on total kcal consumed, with 196 fewer kcal (820 kJ) consumed from the meal chosen from the increased availability menu *v*. the control menu, *F*(1,74) = 46·45, *P* < 0·001, *η* ^2^_p_ = 0·386. There was no main effect of SEP on kcal consumed, *F*(1,74) = 0·179, *P* = 0·673, *η* ^2^_p_ = 0·002, and no interaction between menu type and SEP, *F*(1,74) = 1·580, *P* = 0·213, *η* ^2^_p_ = 0·021, see Fig. 2. The Bayes factor for the main effect of menu type was BF^10^ > 100, indicative of extreme evidence for the alternative hypothesis (i.e. increased availability of lower energy foods decreases energy intake). The Bayes factor for the main effect of SEP was BF^10^ = 0·25, indicative of moderate support for the null hypothesis. Finally, the Bayes factor for the menu*SEP interaction was BF^10^ = 0·30, indicative of moderate support for the null hypothesis.

### Primary analyses (sensitivity)

No participants guessed the primary aim of the study, although a minority (*n* 10/77) believed the study was measuring healthiness of food selected or energy intake. The pattern of results remained the same with these participants excluded. All other sensitivity analyses produced the same pattern of results as in the primary analysis, including when highest education qualification was substituted for other measures of SEP and when order of availability menus was controlled for. See online Supplementary Materials.

### Secondary analyses (total later energy intake)

In our pre-registered analyses, in error we did not specify removal of participants self-reporting implausible or very low later energy intake. Analyses produced the same results with no participants removed, when using the conservative cut-off (*n* 6 (8 %) removed) and using the more stringent cut-off (*n* 25 (32 %) removed). Given that approximately 30–35 % of 24-h energy intake recalls are estimated to be implausible^(36)^, analyses using the more stringent cut-off are reported and the alternative analyses are reported in full in the online Supplementary Materials, see Table 2. Although total later energy intake was somewhat higher after the meal from the increased availability menu (64 kcals/268 kJ), ANOVA revealed no main effect of menu type on later kcal consumed, *F*(1,50) = 0·33, *P* = 0·57, *η* ^2^_p_ = 0·007. There was also no main effect of SEP, *F*(1,50) = 3·51, *P* = 0·067, *η* ^2^_p_ = 0·066, and no interaction between menu and SEP, *F*(1,50) = 0·003, *P* = 0·95, *η* ^2^_p_ < 0·001. The Bayes factor for the main effect of menu was BF^10^ = 0·23, indicative of moderate support for the null hypothesis. The Bayes factor for SEP was BF^10^ = 1·22, indicative of anecdotal support for the alternative hypothesis. Finally, the Bayes factor for the Menu*SEP interaction was BF^10^ = 0·29, indicative of moderate support for the null hypothesis.

**Table 2.** Energy selection and intake (in kcal) by menu type and SEP (Mean values and standard deviations)

	Lower SEP				Higher SEP			
	Control menu		Increased availability menu		Control menu		Increased availability menu	
	Mean	sd	Mean	sd	Mean	sd	Mean	sd
Meal energy								
Total energy of meal selected	956·76	177·03	746·95	230·97	969·45	220·61	694·78	169·77
Total energy intake from meal	809·91	206·65	651·08	270·48	828·40	214·82	597·73	172·60
Later energy intake								
Total energy intake after meal: dataset with stringent cut-offs for implausible energy intake*	1975·15	592	2045·27	625	2328·96	944	2386·27	895
Total energy intake after meal: dataset with conservative cut-offs for implausible energy intake†	1820·51	598·98	1857	644	1893·67	768·9	2066·00	909·56
Total energy intake after meal: full dataset with no removal‡	1784·57	615	1892·14	707	2008·63	958	2257·63	1283

SEP, socio-economic position.

* Values derived from stringent cut-off analysis resulted in an analytic (*n* 52; lower SEP = 26 and higher SEP = 26) after excluding twenty-five participants whose next day energy intake was less than 1000 kcal (female) or 1250 kcal (male).

† Values derived from conservative analysis cut-off analysis resulted in an analytic (*n* 71; lower SEP = 35 and higher SEP = 36) after excluding participants whose next day energy intake was outside of the following ranges: 500–3500 kcal (female) or 800–4200 kcal (male).

‡ Values derived from full dataset with no removal are from an analytic (*n* 77; lower SEP = 37 and higher SEP = 40).

Values are mean and standard deviations for meal energy and later energy intake.

Meal energy refers to energy content of meal items selected and total energy consumed from the meal. Values derived from total energy of meal selected are from an analytic (*n* 77; lower SEP = 37 and higher SEP = 40). Values derived from total energy intake from meal are from an analytic (*n* 76; lower SEP = 37 and higher SEP = 39). Later energy intake refers to self-reported energy intake after the study meal and during the next day.

### Secondary analyses (moderation by individual differences)

There was no evidence that the lower *v*. higher SEP groups differed on any of the individual difference measures and no evidence that individual difference measures moderated the effect of increased availability of lower energy menu options on total meal energy selected or consumed. See online Supplementary Materials for analyses in full.

## Discussion

Changing the availability of lower energy ready meal food options (main meal and side dish) from 30 % (standard availability) to 70 % resulted in participants consuming 196 fewer kcal (820 kJ) during an evening meal. Subsequent energy intake that evening and the next day was somewhat higher in the increased *v*. standard availability condition (+64 kcal/268 kJ), but this difference was small and not statistically significant. There was no evidence that the effects of increasing lower energy food options were moderated by SEP, indicating that this intervention approach – on immediate and later energy intake – is likely to have equitable effects for those with lower and higher SEP. These findings are in line with suggestions that increasing the availability of lower energy food options is a potentially powerful and equitable approach to improving diet^(7)^.

No studies we are aware of have examined whether interventions designed to increase the availability of lower energy meals alter subsequent eating behaviour. It is well established from laboratory appetite experiments that reductions to energy intake are in part compensated for later in the day by eating more. For example, recent meta-analyses examining the impact that serving food with a lower energy content at a meal estimate that between 11 and 42 % of reduced energy intake at that meal is compensated for^(15,16)^. In the present study, this figure equated to 33 %. Critically, we found no evidence that this degree of compensation differed between participants of higher and lower SEP. Furthermore, the effects of increased availability on energy intake were not dependent on a range of participant characteristics (e.g. results were similar in participants who reported being more *v*. less motivated by health or weight control when making day-to-day food choices).

A better understanding of why increasing the availability of lower energy options decreases energy intake may inform attempts to identify when and for whom availability interventions will be of most benefit. In line with Pechey and colleagues^(37)^, we presume that effects are largely explained by the observation that under conditions of lower availability, the probability of a lower energy option being highly preferred is markedly reduced than under conditions of increased availability. More direct testing of this proposed mechanism would now be informative as it suggests that if lower energy options selected to replace higher energy options are similarly liked across population subgroups, increasing the availability of lower energy food options would be a socially equitable dietary intervention.

A strength of the present study is that it is the first we are aware of to examine longer-term effects on energy intake of increasing availability of healthier foods in real-world settings, as opposed to hypothetical experiments^(12)^ or real-world studies unable to quantify participant-level energy intake^(38)^. We examined energy intake for 24 h, and it is plausible that greater compensation would occur over longer time frames. However, findings on energy intake compensation over time suggest that any increase in compensation over time would likely be small^(15,16)^. The experimental design allowed us to isolate the independent effect of increasing availability of lower energy options, but in doing so, price was held constant across availability conditions. Cost motivates food choices, and therefore it would be informative to examine whether the present findings would be replicated when participants have to pay for their own meals. Further, while cost was held constant (price information was not provided and participants did not pay for their meals), the average retail price of higher energy options was slightly more expensive than the lower energy options offered. It is possible that obtaining the more expensive items for free would be perceived as a greater gain than obtaining the less expensive items. However, this would be contingent on participants being aware of the true price of the food items on offer, and we presume this would be unlikely. In a survey of over 3000 individuals, half responded that they eat ready meals at least weekly; in general, the prevalence of ready meal consumption in the UK is high with an estimated 79 million ready meals consumed in the UK each week^(39)^. The experimental paradigm meant participants ordered single-serve ready meals, so it is unclear whether these findings would apply to ready meals which serve groups of individuals (e.g. families eating together). To be able to examine food consumption in real-world settings, we relied on participants photographing their meals and reporting on their energy intake. Self-reported dietary data are prone to bias and may be larger in participants of lower *v*. higher SEP^(40)^. However, compliance with study instructions was high, and sensitivity analyses accounted for implausible/improbable dietary reporting. Because SEP is multi-faceted, a further strength of the present study is that results were consistent when using a range of SEP indices (i.e. education level, household income, subjective social status). As the sample was predominantly White, future research of more ethnically diverse samples and implementing availability interventions in real-life settings to examine longer-term changes to energy intake and body weight would now be informative. Finally, the present study was not powered to detect very small effects (such as the interaction between availability and SEP). However, Bayesian analyses of the availability manipulation and SEP interaction effect indicated support for the null (evidence of absence), as opposed to a meaningful interaction effect that was not detectable due to a lack of statistical power (absence of evidence)^(41)^.

### Conclusions

Increasing the availability of lower energy evening meal food options in online food ordering decreases energy intake, and the impact that availability had on meal and subsequent 24-h energy intake was similar in participants of lower and higher SEP. Increasing the availability of lower energy food options is likely to be an effective and equitable public health approach to improving diet.

## Supplementary material

For supplementary material accompanying this paper visit https://doi.org/10.1017/S0007114522002197. click here to view supplementary material
