# Supplementary material for: The impact of increasing the availability of lower energy foods for home delivery and socio-economic position: a randomised control trial examining effects on meal energy intake and later energy intake
Source: Br J Nutr. 2022 Aug 1;129(7):1280–8. doi: 10.1017/S0007114522002197 (PMC10011593; doi:10.1017/S0007114522002197)
Supplement: Supplementary file 1 [file S0007114522002197sup001.pdf]

Online supplementary materials for Langfield et al. Socioeconomic position and the impact of increasing the availability of lower energy foods for home delivery: a randomized control trial examining effects on meal energy intake and later energy intake

1. Menu Information

		Food item	Energy content (kcals)	Category of item
Control Menu	Mains	Sausage & Mash	714	Higher energy
		Cod Mornay	335	Lower energy
		Beef Stroganoff	663	Higher energy
		Sausage Pasta	780	Higher energy
		Macaroni Cheese	705	Higher energy
		Irish Stew	196	Lower energy
		Chicken Jambalaya	319	Lower energy
		Pulled beef	580	Higher energy
		Chicken Black Bean	526	Higher energy
		Spaghetti Bolognese	555	Higher energy
	Sides	Chunky Chips	546	Higher energy
		Red Cabbage	236	Lower energy
		Roast Potatoes	512	Higher energy
		Carrot and Swede mash	360	Higher energy
		Potato Dauphinoise	504	Higher energy
		Carrot Sweetcorn Peas	256	Lower energy
Increased Availability Menu	Mains	Cod Mornay	335	Lower energy
		Shepherd’s Pie	339	Lower energy
		Prawn Linguine	318	Lower energy
		Sausage Pasta	780	Higher energy
		Chicken Jambalaya	319	Lower energy
		Spaghetti Bolognese	555	Higher energy
		Irish Stew	196	Lower energy
		Mushroom Risotto	339	Lower energy
		Chicken in Mushroom sauce	281	Lower energy
		Chicken Black Bean	526	Higher energy
	Sides	Carrot Sweetcorn Peas	256	Lower energy
		Peas and Carrots	242	Lower energy
		Carrot and Swede mash	360	Higher energy
		Chunky Chips	546	Higher energy
		Red Cabbage	236	Lower energy
		Cauliflower Cheese	258	Lower energy

Supplementary Table S1: Menu Item Kcal Information

Note. Categories of items also denoted by shading - green shading reflects lower energy foods and red shading reflects higher energy foods.

Supplementary Table S2: Menu Option Liking Ratings and Requested Nutritional Information

	**Lower SEP (n = 37)**	**Higher SEP (n = 40)**	**Overall (N = 77)**
**Main meal food ratings ("how much would you like this item?")**			
Sausage & Mash	4.89 (1.52)	4.50 (1.72)	4.69 (1.63)
Cod Mornay	3.32 (1.91)	3.78 (2.18)	3.56 (2.06)
Beef Stroganoff	3.95 (1.68)	4.53 (1.92)	4.25 (1.82)
Sausage Pasta	4.41 (1.88)	4.35 (1.79)	4.38 (1.82)
Macaroni Cheese	3.81 (2.21)	4.40 (1.78)	4.12 (2.01)
Irish Stew	4.32 (1.89)	4.40 (1.88)	4.36 (1.87)
Chicken Jambalaya	4.78 (1.96)	4.50 (1.70)	4.64 (1.82)
Pulled beef	4.86 (2.06)	4.90 (1.78)	4.88 (1.91)
Chicken Black Bean	4.92 (1.99)	4.97 (1.56)	4.95 (1.77)
Spaghetti Bolognese	4.81 (1.76)	4.90 (1.55)	4.86 (1.64)
Prawn Linguine	3.51 (2.16)	3.87 (2.49)	3.70 (2.32)
Shepherd's Pie	4.84 (1.72)	4.85 (1.86)	4.84 (1.79)
Chicken in Mushroom sauce	4.22 (1.99)	4.10 (2.07)	4.16 (2.02)
Mushroom Risotto	3.68 (2.07)	3.80 (2.27)	3.74 (2.16)
**Side dish food ratings ("how much would you like this item?")**			
Chunky Chips	6.24 (1.21)	6.07 (1.35)	6.16 (1.28)
Carrot Sweetcorn Peas	4.76 (1.89)	4.68 (1.76)	4.71 (1.81)
Peas and Carrots	4.86 (1.81)	4.70 (1.79)	4.78 (1.79)
Potato Dauphinoise	4.97 (1.82)	5.78 (1.63)	5.39 (1.76)
Roast Potatoes	6.08 (1.04)	5.90 (1.41)	5.99 (1.24)
Red Cabbage	3.97 (2.13)	4.00 (2.12)	3.99 (2.11)
Carrot and Swede mash	5.24 (1.79)	5.00 (1.68)	5.12 (1.72)
Cauliflower Cheese	4.54 (2.33)	4.83 (2.17)	4.69 (2.24)
**Requested nutritional information about the dishes on each menu (N/% yes)**			
Control menu mains	2 (2.6%)	10 (13.0%)	12 (15.6%)
Control menu sides	2 (2.6%)	8 (10.4%)	10 (13.0%)
Increased availability menu mains	2 (2.6%)	8 (10.4%)	10 (13.0%)
Increased availability menu sides	1 (1.3%)	7 (9.1%)	8 (10.4%)

Note. Values are mean (standard deviation) or number of participants (%). Green shading reflects lower energy foods and red shading reflects higher energy foods.

<u>Menu Liking Rating Analyses</u>

A mixed ANOVA was used to test the within-subjects effect of food category (lower energy vs higher energy), the between-subjects effect of SEP (lower vs higher educational qualification), and the interaction between food category*SEP on mean liking. There was a main effect of food category $F(1,75) = 61.76$, $p < .001$, $\eta 2_p = .452$, with lower rated liking for lower energy foods (EMM = 4.29, 95% CI: 4.04, 4.53) than higher energy foods (EMM = 4.98, 95% CI: 4.77, 5.18), although both were liked. There was no main effect of SEP or interaction between SEP and food category on mean liking (ps > .70, $\eta 2_p$s < .002). To further test whether differences in liking for higher/lower energy foods translated into differences in liking for the control vs increased availability menu, a mixed ANOVA was used to test the within-subjects effects of menu type (control vs. increased availability), the between-subject effect of SEP (lower vs higher educational qualification), and the interaction between menu*SEP on mean liking. There was a main effect of menu $F(1,75) = 20.26$, $p < .001$, $\eta 2_p = .213$, with lower rated liking for the increased availability menu (EMM = 4.53, 95% CI: 4.32, 4.76) than the control menu (EMM = 4.75, 95% CI: 4.54, 4.96), although both were well liked. There was no main effect of SEP or interaction between SEP and menu on mean liking (ps > .54, $\eta 2_p$s < .005).

Supplementary Figure S1. *Control (top panel) and Increased Availability (bottom panel) menus with lower energy mains and sides highlighted in green*

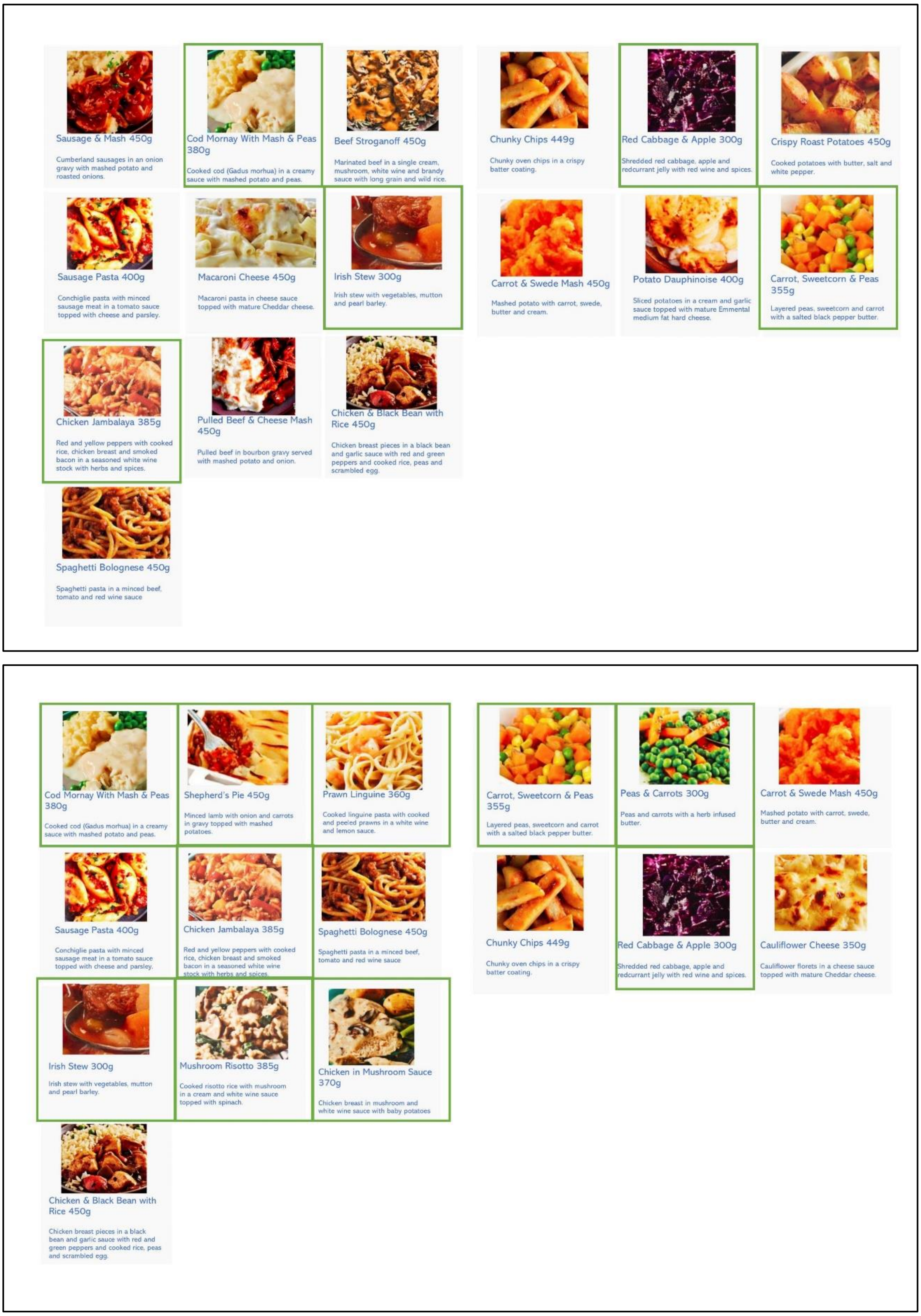

Supplementary Figure S2. *Online food ordering platform as shown to participants*

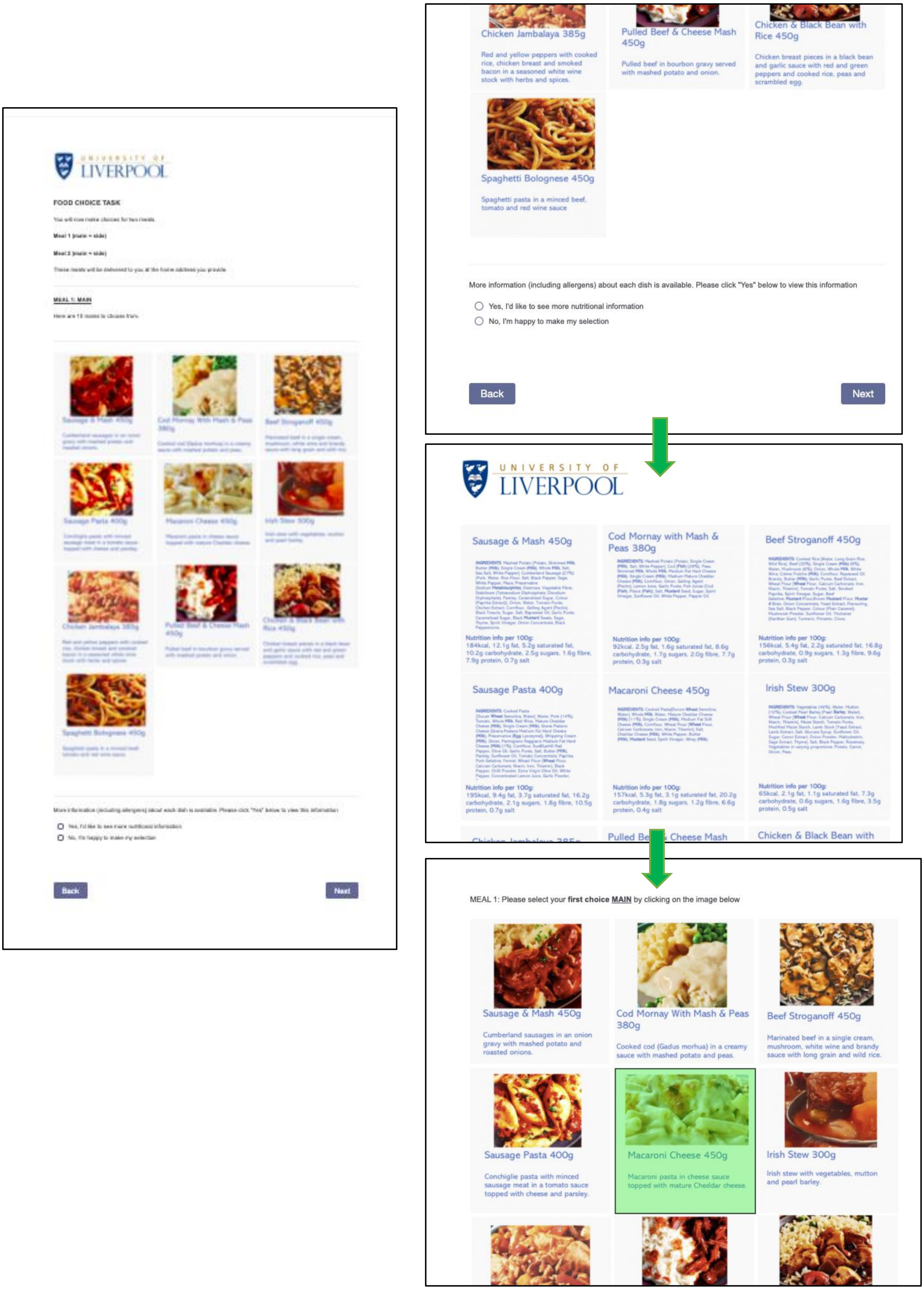

2. Participant Measures

2.1 Highest educational qualification

The primary measure of SEP in this study was highest educational qualification, coded from 1 to 9 (1 = No formal qualifications; 2 = 1–3 GCSEs; 3 = 4+ GCSEs; 4 = A level; 5 = Certificate of higher education (CertHE); 6 = Diploma of higher education (DipHE); 7 = Bachelor; 8 = Master's degree; 9 = Doctorate). This was categorised as a binary variable: "lower" (values: 1, 2, 3, 4; A level/equivalent or less) or "higher" (values: 5, 6, 7, 8, 9; qualifications beyond A level).

2.2. Additional measures of SEP

A composite score of level of education was created (highest educational qualification and number of years in higher education z-scored and averaged). Number of years in higher education was measured by asking participants '*After leaving school (i.e. at 16 years old), how many further years of higher education (i.e. a formal course) did you study for? If you left school and did not go on to study further in higher education, your answer would be 0. If you left school and then studied for two years for A levels, your answer would be 2. If you completed A levels over two years and then also studied for a three year undergraduate degree, your answer would be 5.*'.

Equivalised disposable income was calculated from the question asking participants '*What is your annual after tax household income, including all earners in your household, in GBP (to the nearest £1000)? (range £ 0 - 999,999)*' and participants also reported on household composition ('*Thinking about all of the people who live at your house, including you: How many adult(s) or children aged 14 and over live at your house? How many child(ren) under the age of 14 live at your house?*'). The OECD-modified equivalence scale was used to adjust household income taking into account household size and composition. Equivalised household income was calculated by dividing the after-tax household (including all earners to the nearest ¬£1000) by the sum of the equivalence value of all the household members (1 = first adult; 0.5 = additional adult or child >14 years old; 0.3 = child aged 0-13 years old).

Subjective social status (SSS) was assessed the MacArthur Scale, rated from 1 (lower SSS) – 10 (higher SSS).

2.3 Demographic and personal characteristics

We measured gender, age, ethnic group, employment/student status, dieting status (Yes/no), physical activity level (number of days in the last week), and ready meal consumption frequency ("never or not in the last year", "less than once per month", "1-3 times per month", "1-2 times per week", "3 times per week or more"). We also assessed BMI calculated in $kg/^{m2}$ from participant self-reported weight and height.

3. Study Sample Size

A recent review suggested increasing availability of heathier options may reduce food intake by 17-36% (Hollands et al., 2019). To be conservative, and in the context of the menus designed in the current study (energy content of the food items on Menu 1 and Menu 2 differs by ~ 120kcal; SD = 181kcal), we powered the study to detect a difference of 60kcal, or 14% (based on the mean number of menu kcals for main and side combined), on outcome measures, assuming SD = 181kcal, giving f = .17. Sample size calculations were performed in G*Power. Assuming 80% power, with alpha set at 5%, to detect a main effect of

proportion with an effect size of *f* = .17, with 2 groups and 2 measurements, and correlation among repeated measures set at 0.5, a sample of 70 was required. For the within-between interaction (SEP: higher v lower) x (menu type: control v increased availability), based on previous findings (e.g. Marty et al., 2020), we hypothesised moderation by SEP is unlikely. We therefore powered this analysis to detect the same effect size as above of *f* = .17, also giving a sample of 70 participants would be required. Given likely attrition due to of logistical concerns (missing or incorrect items being received), missing image or diary data, or participants failing consistency/attention checks, we aimed to recruit a total of 88 participants that completed the study.

4. <u>Results</u>

4.1 Missing data.

Of the n = 77 analysed, for primary and secondary outcome measures there was 98.7% complete data, with 1.3% missing on total kcal consumed (due to unclear photographs of the meal meaning it was too difficult to extract kcal consumed) for the control meal, and 100% complete data on all other primary or secondary outcome measures. Given the small amount of missing data, multiple imputation was not conducted, and data was analysed on complete cases only for total kcal consumed (i.e. N = 76). All other analyses involved the full sample N = 77.

4.2 Impact of menu type and SEP on kcal selected

A mixed ANOVA was conducted to examine the within-subject effect of menu type (control vs. increased availability), the between-subject effect of SEP (higher vs lower), and the interaction menu*SEP on total kcal selected. The ANOVA revealed a main effect of menu type on total kcal selected, with fewer kcal selected from the increased availability menu vs the control menu, $F(1,75) = 66.04$, $p < .001$, $\eta 2_p = .47$. There was no main effect of SEP on total kcal selected, $F(1,75) = 0.32$, $p =.57$, $\eta 2_p = .004$, and no interaction between Menu and SEP on total kcal selected, $F(1,75) = 1.18$, $p = .28$, $\eta 2_p = .016$. The Bayes factor for the main effect of menu type was BF10 > 100, indicative of extreme evidence for the alternative hypothesis. The Bayes factor for the main effect of SEP was BF10 = 0.24, indicative of moderate support for the null hypothesis. Finally, the Bayes factor for the menu * SEP interaction was BF10 = 0.41, indicative of anecdotal support for the null hypothesis.

Supplementary Figure S3*: Meal kcal selected by menu condition and SEP*

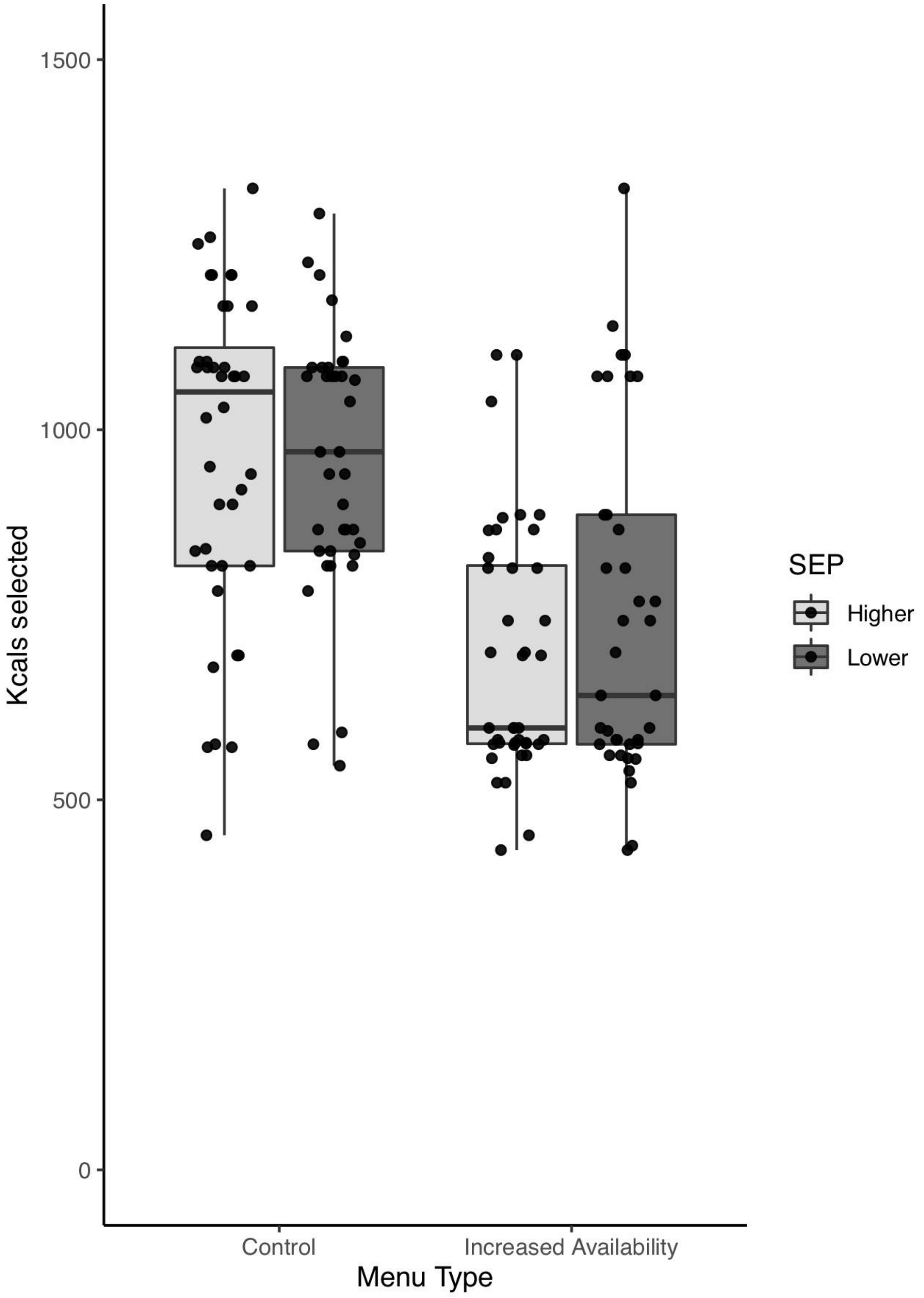

4.3 Sensitivity Analyses for Primary Outcomes

4.3.1 Alternative measures of SEP

To assess the robustness of the primary findings, the analyses were repeated (n = 77), substituting SEP (higher education level, lower education level) with other measures of SEP collected in this study: level of education (number of years in higher education plus qualifications achieved), equivalised household income, and subjective socioeconomic status. Results were consistent with the primary analysis when substituting SEP with level of education predicting total kcal selected and total kcal consumed; there were main effects of menu (*p*s < .001, $\eta 2_p$s >.387), level of education did not predict total kcal selected nor consumed (*p*s > .333, $\eta 2_p$s < .013), and there were no interactions (*p*s > .28, $\eta 2_p$s < .016). Results were also consistent with the primary analysis when substituting SEP with equivalised household income predicting total kcal selected and total kcal consumed; there were main effects of menu (*p*s < .001, $\eta 2_p$s > .219), income did not predict total kcal selected nor consumed (*p*s > .561, $\eta 2_p$s < .005), and there were no interactions (*p*s > .148, $\eta 2_p$s < .028). Results were largely consistent when substituting SEP with SSS predicting total kcal selected and total kcal consumed; though there was no longer a main effect of menu on total kcal selected (p = .06, $\eta 2_p$ = .047), there was a main effect of menu on total kcal consumed (p = .004, $\eta 2_p$ = .108), SSS did not predict total kcal selected nor consumed (*p*s > .500, $\eta 2_p$s < .006), and there were no interactions (*p*s > .185, $\eta 2_p$s < .024).

4.3.2 Removing study aim guessers

The primary analyses were repeated after removing participants who correctly identified the aims of the study (n = 0) or the aim of the study outcome measures (i.e. to measure the healthiness of their food selection, calories consumed, how much was eaten etc; n = 10), leaving a total sample for this sensitivity analysis of n = 67 for total kcal selected and n = 66 for total kcal consumed. The results of these analyses were consistent with the primary analysis on total kcal selected and total kcal consumed; there were main effects of menu (*p*s <.001; $\eta 2_p$s > .393), no main effects of SEP (*p*s > .635, $\eta 2_p$s < .003), and no interactions (*p*s > .10, $\eta 2_p$s < .041).

4.3.3 Retaining participants who did not adhere to the study instructions

The primary analyses were repeated after retaining those who were excluded for not following the study procedures, leaving the full sample of completers for this sensitivity analysis of n = 88 for total kcal selected and n = 87 for total kcal consumed. The results of these analyses were consistent with the primary analyses on total kcal selected and total kcal consumed; there were main effects of menu (*p*s < .001, $\eta 2_p$s > .371), no main effects of SEP (*p*s > .792, $\eta 2_p$s < .001), and no interactions (*p*s > .152, $\eta 2_p$s < .024).

4.3.4 Excluding participants who received incorrect items

The primary analyses were repeated after removing participants who received items that differed in cuisine or category (e.g. lower energy item rather than higher energy item; n = 6) and participants who received items which they rated poorly (n = 2), leaving a total sample for the sensitivity analysis of n = 69 for total kcal selected and n = 68 for total kcal consumed. The results of these analyses were consistent with the primary analysis on total kcal selected and total kcal consumed; there were main effects of menu (*p*s < .001; $\eta 2_p$s > .388), no main effects of SEP (*p*s > .548; $\eta 2_p$s < .005), and no interactions (*p*s > .208; $\eta 2_p$s < .039).

4.3.5 Controlling for order effects

The primary analyses were repeated adjusting for order menus presented (Control Menu first vs Increased Availability Menu first) and order meals consumed (Control Meal first vs Increased Availability Meal first) as a between-subjects variable in the model. Although participants were asked to eat the meals in the same order as they were shown the menus, n = 2 ate them the wrong way around. A 2 (Control Menu first vs Increased Availability Menu first) x 2 (Control Menu vs Increased Availability Menu) x 2 (Lower SEP vs Higher SEP) mixed ANOVA revealed a main effect of Menu ($p < .001$, $\eta 2_p = .468$), no order effect, no effect of SEP, and no interactions (*p*s> .290, $\eta 2_p$s < .015), on total kcal selected. A 2 (Control Meal first vs Increased Availability Meal first) x 2 (Control Meal vs Increased Availability Meal) x 2 (Lower SEP vs Higher SEP) mixed ANOVA also revealed a main effect of Menu ($p < .001$, $\eta 2_p = .396$), no order effect, no effect of SEP, and no interactions (*p*s> .145, $\eta 2_p$s < .029), on total kcal consumed.

4.4. Impact of menu and SEP on later energy consumed

4.4.1 No participants removed

A mixed ANOVA was conducted to examine the within-subject effect of Menu (Control vs Increased Availability), the between-subject effect of SEP (higher vs lower), and the interaction Menu*SEP on compensatory kcal consumed (self-reported kcal consumed after the study meal until midnight the following night). Although compensatory kcal consumed was higher after the Increased Availability meal, the ANOVA revealed no main effect of Menu on compensatory kcal consumed, $F(1,75) = 1.78$, $p = .19$, $\eta 2_p = .023$. The pattern of findings indicated that lower SEP individuals reported fewer kcal consumed after the study meal, though there was no statistical evidence of a main effect of SEP on compensatory kcal consumed, $F(1,75) = 3.13$, $p = .081$, $\eta 2_p = .04$, and no interaction between Menu and SEP on compensatory kcal consumed, $F(1,75) = 0.28$, $p = .60$, $\eta 2_p = .004$.

4.4.2 Conservative sensitivity analysis

Implausible daily calorie intake values were defined as outside of the following ranges: 500-3500kcal (females) and 800-4200kcal (males), with standardised/crude cut offs based on a review of previous research. The pattern of findings was consistent with the main secondary analysis, with no main effect of Menu, SEP and no interaction Menu*SEP (*p*s > .320).

4.5 Individual difference measures and the effect of menu type on kcal selected/consumed

There was no evidence of a difference between higher SEP and lower SEP individuals on food choice motives around health (t(75) = 0.644, $p = .521$, $d = .146$), food choice motives around weight control ($t(75) = 1.371$, $p = .174$, $d = .313$), satiety responsiveness ($t(75) = -0.532$, $p = .596$, $d = .121$), plate clearing tendencies ($t(75) = -0.068$, $p = .946$, $d = .016$), or food waste concern ($t(75) = 0.031$, $p = .976$, $d = .007$). Five mixed ANOVA were conducted predicting meal energy selected, examining the within-subject effect of menu type, each individual difference measure as a covariate, and the interaction between menu type and each individual difference measure. The ANOVA on the effect of menu type, food waste concerns and the interaction revealed that higher food waste concern predicted a greater number of kcal selected F(1,75) = 7.68, $p = .007$, $\eta 2_p = .093$. This model also revealed that fewer kcal were selected from the increased availability menu, F(1,75) = 15.41, p <.001, $\eta 2_p = .170$, though there was no evidence of an interaction ($p = .045$, $\eta 2_p = .053$). The other four ANOVA revealed no main effects of menu type (*p*s > .015, $\eta 2_p$s < .077), no main effect of the individual difference measures (*p*s > .389, $\eta 2_p$s < .010), and no interactions between the individual difference measure and menu type predicting total kcal selected (*p*s > .404, $\eta 2_p$s <

.009). Five mixed ANOVA were conducted predicting total meal energy consumed, examining the within-subject effect of menu type, each individual difference measure as a covariate, and the interaction between menu type and each individual difference measure. The two ANOVA on the effects of food choice motives (health, weight control) found no evidence that food choice motives predicted total meal energy consumed (*p*s >.565, $\eta 2_p$s = .004), a main effect of menu when adjusting for FCM health (*p* = .009, η2p = .089), no main effect of menu when adjusting for FCM weight control (*p* =.112, $\eta 2_p$ =.034), and no interactions between menu and food choice motives (*p*s >.291, $\eta 2_p$ < .015). In the remaining three ANOVAs, each of the three models revealed that the individual difference measure predicted total meal energy consumed, with increased kcal consumed predicted by higher satiety responsiveness (F(1,74) = 10.12, *p* = .002, $\eta 2_p$ = .119), increased plate clearing tendencies (F(1,74) = 15.68, *p* < .001, $\eta 2_p$ = .173), and increased food waste concern (F(1,74) = 16.09, *p* < .001, $\eta 2_p$ = .177). The model including satiety responsiveness found a main effect of menu type (F(1,74) = 7.11, *p* = .009, $\eta 2_p$ = .088), as did the model including food waste concern (F(1,74) = 10.30, *p* = .002, $\eta 2_p$ = .122), but the model including plate clearing did not (*p* = .039, $\eta 2_p$ = .056), and no interactions were observed between the individual difference measures and menu type (*p*s > .105, $\eta 2_p$s < .035).

Supplementary Table S3: Individual difference measures, split by SEP group and overall

	Lower SEP (n = 37)	Higher SEP (n = 40)	Overall (N = 77)
**Food choice motives**			
Heath	2.93 (0.795)	2.82 (0.662)	2.87 (0.726)
Weight control	2.84 (0.874)	2.57 (0.861)	2.70 (0.872)
**Satiety Responsiveness**	2.22 (0.952)	2.33 (0.840)	2.27 (0.891)
**Plate clearing tendencies**	3.96 (0.862)	3.97 (0.842)	3.96 (0.846)
**Food waste concerns**	3.41 (0.842)	3.41 (0.815)	3.41 (0.822)

Notes. Values are means (standard deviation). Higher scale score indicate greater endorsement; food choice motives scales: health, weight control (1-5), satiety responsiveness scale (1-5), plate clearing tendencies scale (1-5) and food waste concerns scale (1-5)

5. References

Hollands, G. J., Carter, P., Anwer, S., King, S. E., Jebb, S. A., Ogilvie, D., ... & Marteau, T. M. (2019). Altering the availability or proximity of food, alcohol, and tobacco products to change their selection and consumption. *Cochrane Database of Systematic Reviews*, (9).

Marty, L., Jones, A., & Robinson, E. (2020). Socioeconomic position and the impact of increasing availability of lower energy meals vs. menu energy labelling on food choice: two randomized controlled trials in a virtual fast-food restaurant. *International Journal of Behavioral Nutrition and Physical Activity*, *17*(1), 1-11.
